# Absolute Cross-Section Measurements in XQQ Instruments: The NBS Round Robin

**DOI:** 10.6028/jres.093.106

**Published:** 1988-06-01

**Authors:** Richard I. Martinez

**Affiliations:** Center for Chemical Physics, National Bureau of Standards, Gaithersburg, MD 20899

Triple-quadrupole (QQQ) tandem mass spectrometry (MS/MS) is used for the analysis of multicomponent mixtures [1]. The analysis makes use of the collisionally activated dissociation (CAD) of “parent” ions. A “parent” ion may be a molecular radical cation, a protonated molecule, or a “progeny” fragment ion (daughter, granddaughter, etc., produced by the CAD of a larger precursor parent ion). A “parent” ion selected by the first quadrupole (Q1) interacts with a target gas within the second quadrupole (Q2). Q2 channels undissociated “parent” ions and “progeny” fragment ions into the third quadrupole (Q3) for mass analysis. The instrument thus produces a CAD spectrum of each initially selected “parent” ion.

But XQQ instruments (QQQ, BEQQ, etc.) are complex ion-optical devices [2–8]. So the choice of parameter settings and/or of instrument design can provide a distorted view of the molecular dynamics of the CAD process (e.g., if there are scattering losses due to poor ion containment within Q2, fringing fields between Q2/Q3, etc. [2–8]). So one observes instrument-*dependent* CAD spectra.

The key MS/MS parameters are:

1. the “target thickness” = (actual path length traversed by the ion in its complex oscillatory trajectory through the gas target) × (effective number density of the CAD target gas);
2. the type of target gas (influences the extent of energy transfer);
3. the center-of-mass interaction energy, *E*_cm_;
4. the energy level of the analyzing quadrupole Q3 relative to that of Q2;
5. the Mathieu parameter *q*_2_ (rf voltage of Q2) and restrictive interquadrupole apertures of diameter <1.4 *r*_0_; and
6. differences in mass-dependent conversion gain of ion detectors.

For any *one* molecule, these key MS/MS parameters can cause the relative intensities among its various progeny ions to differ significantly when measured in different XQQ instruments.

This was clearly demonstrated in a recent international round robin [9] wherein very *different CAD spectra were observed for the same molecule*. That is, the relative intensities measured in different QQQ instruments for any given pair of progeny ions differed by factors ranging into the hundreds, even though the same *nominal* operating conditions were supposedly used in each of the QQQ instruments. So a CAD spectrum of a given species in one XQQ instrument presently cannot be used to identify and quantitate that same species in a different XQQ instrument. Moreover, real-time trace analysis often involves unknown transient species (e.g., reactive environmental pollutants and/or products of their photooxidation). Characterization of such species would be facilitated if one could do spectral matching against a generic, instrument-independent MS/MS CAD spectral database comprised of *known* ionic substructures (see [10,11]).

To obtain standardized instrument-*independent* CAD spectra, one must make appropriate corrections for ion-optical effects within each XQQ structure. To do so, one must provide a dynamically correct basis for selecting the key MS/MS parameter settings within each XQQ instrument.

The reactivity (kinetics) of a molecular system is an intrinsic and generic (transferable) property of that system. So the kinetics (reaction mechanisms and rate coefficients) of selected ion-molecule reactions can be used as molecular probes to determine which combination of key MS/MS parameters provide dynamically correct branching ratios for the CAD of polyatomic ions. To do so, all measurements must be made under pseudo-first order, single-collision conditions such that the following kinetic relations are applicable within a reaction zone of length *L*.

$$\begin{array}{rr} {\text{For A}}^{+}+B\to C^{+}+S & \;\left[\alpha \sigma \right]\\ \to D^{+}+T & \left[\beta \sigma \right]\\ \;\text{etc}. \end{array}$$

(*σ* = total cross-section; branching ratios *α+β+γ+* … = 1),

$$\ln Y\equiv \ln\left\{{\left[A^{+}\right]}_{0}/\left[A^{+}\right]\right\}=\sigma \left[B\right]L$$ (1)

and

$$\begin{array}{c} \;\ln W_{\alpha }\equiv \ln\left\{\alpha {\left[A^{+}\right]}_{0}/\left(\alpha {\left[A^{+}\right]}_{0}-\left[C^{+}\right]\right)\right\}\\ =\sigma \left[B\right]L \end{array}$$ (2)

$$\begin{array}{l} \ln W_{\beta }\equiv \ln\left\{\beta {\left[A^{+}\right]}_{0}/\left(\beta {\left[A^{+}\right]}_{0}-\left[D^{+}\right]\right)\right\}\\ \;=\sigma \left[B\right]L\\ \text{etc}. \end{array}$$ (3)

Equations (1)–(3), etc., place severe constraints [11] on the selection of the key MS/MS parameter settings. The key requirement, however, is that the XQQ instrument must have a *dynamically correct* design. That is, the design should make it possible to adequately control the key MS/MS parameters to provide an undistorted (unbiased) representation of the CAD dynamics (i.e., dynamically correct branching ratios, no back reactions, no impurity reactions, no scattering losses, minimal fringing fields, no mass discrimination, well-defined gas target, etc.). Instrument designs which are incompatible with these requirements cannot provide dynamically correct performance. A dynamically correct instrument is kinetically well behaved if the *σ* derived from eq (1) equals the *σ* from eq (2) for a charge transfer reaction with *α*=1. Charge transfer reactions are dynamically equivalent to a “worst-case” CAD reaction system because they take place at large impact parameters with nearzero momentum transfer.

Work from this lab [10,12–14] has demonstrated that dynamically correct branching ratios *α, β, …*, etc., can be measured in our kinetically well-behaved QQQ instrument [15] when the key MS/MS parameters are properly selected. For N_2_^+^(sf_6_,n_2_)sf_x_^+^(x=1–5), *α*+*β*+*γ*+*δ*+*ϵ*≃1.0 when *σ* derived from eq (1) equals *σ* from eq (2), (3), etc. [14].

Our *σ* values for Ar^+^(Ar,Ar)Ar^+^ [10] and Ne^+^(Ne,Ne)Ne^+^ [12] agreed to within ±10% with experimental and theoretical results from the literature. So the largest *absolute* uncertainty in the effective target thickness {[B]*L*}, and therefore in our *σ* values is probably on the order of ±10%. This ±10% error estimate is consistent with our observations that deviations from linearity in In *W* vs *P* plots occurred only when single-collision conditions were exceeded. So single-collision conditions must be used if one is to develop and use a generic, instrument-independent MS/MS CAD spectral database [10,11].

The NBS Round Robin will provide a “zeroth-order” assessment about which XQQ instruments have dynamically correct designs, and therefore may be well suited for the generation of standardized reference CAD spectra. A test protocol is being formulated. It will involve: i) the in-situ target thickness calibration of each participant’s XQQ instrument {by using eqs (1) and (2) with our *σ* for Ar^+^(Ar,Ar)Ar^+^ [10] to determine the target thickness [B]*L*}, followed by ii) the experiment associated with figure 1(a) of [9].
